# Recent Developments and Applications of Tactile Sensors with Biomimetic Microstructures

**DOI:** 10.3390/biomimetics10030147

**Published:** 2025-02-27

**Authors:** Fengchang Huang, Xidi Sun, Qiaosheng Xu, Wen Cheng, Yi Shi, Lijia Pan

**Affiliations:** 1 Collaborative Innovation Center of Advanced Microstructures, School of Electronic Science and Engineering, Nanjing University, Nanjing 210093, China; 2 School of Integrated Circuits, Nanjing University, Suzhou 215163, China

**Keywords:** bioinspired design, biomimetic tactile sensor, texture recognition, human health detection, human–machine interaction, flexible electronics

## Abstract

Humans possess an innate ability to perceive a wide range of objects through touch, which allows them to interact effectively with their surroundings. Similarly, tactile perception in artificial sensory systems enables the acquisition of object properties, human physiological signals, and environmental information. Biomimetic tactile sensors, as an emerging sensing technology, draw inspiration from biological systems and exhibit high sensitivity, rapid response, multimodal perception, and stability. By mimicking biological mechanisms and microstructures, these sensors achieve precise detection of mechanical signals, thereby paving the way for advancements in tactile sensing applications. This review provides an overview of key sensing mechanisms, microstructure designs, and advanced fabrication techniques of biomimetic tactile sensors. The system architecture design of biomimetic tactile sensing systems is also explored. Furthermore, the review highlights significant applications of these sensors in recent years, including texture recognition, human health detection, and human–machine interaction. Finally, the key challenges and future development prospects related to biomimetic tactile sensors are discussed.

## 1. Introduction

With the rapid advancement of science and technology, tactile sensors have emerged as a pivotal driver in advancing wearable electronics [1,2,3,4,5]. Compared to traditional rigid sensors, flexible tactile sensors offer significant advantages for artificial tactile systems. These benefits include their adaptability to complex surfaces, improved sensitivity, and the ability to mimic human-like tactile perception [6,7,8,9,10]. These sensors enable the detection of a wide range of physical signals, including pressure, temperature, and vibration [11,12,13,14]. This capability allows for precise identification of surface characteristics and facilitates rapid adaptation to environmental changes [15,16]. For example, in human–machine interaction (HMI), flexible tactile sensors enhance natural and intuitive user interfaces, enabling users to interact more effectively with devices through touch [17,18]. In robotics, these sensors improve robots’ tactile abilities, facilitating better task execution in complex environments, such as grasping and manipulating objects [19,20]. Furthermore, flexible tactile sensors are crucial in health monitoring, as they continuously monitor physiological signals like heart rate and blood pressure, enabling early diagnosis and treatment of diseases [21,22].

In recent years, there has been significant development in various flexible tactile sensors based on different sensing mechanisms, such as piezoelectric, capacitive, piezoresistive, and triboelectric sensors [23,24,25,26]. Among these, sensors with biomimetic microstructures stand out, due to their high sensitivity [27,28], rapid response [29,30], continuous tactile data acquisition [31,32], multimodal perception [33,34], and high spatial resolution [20,35].

**Figure 1:**
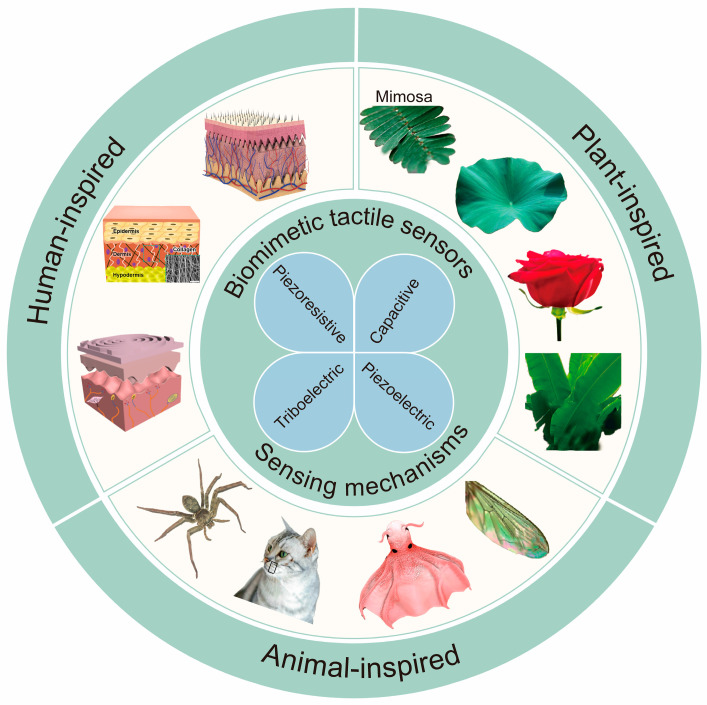
Overview of biomimetic tactile sensors. Clockwise from Mimosa picture [36]: copyright 2014, John Wiley and Sons—lotus leaf [37]: copyright 2011, Beilstein Institute; rose petals [38]: copyright 2024, Elsevier; banana leaf [39]: copyright 2017, American Chemical Society; cicada wing [40]: copyright 2023, American Chemical Society; glowing sucker octopus [41]: copyright 2022, John Wiley and Sons; cat whisker [42]: copyright 2023, Springer Nature; spider [43]: copyright 2023, The American Association for the Advancement of Science; fingertip structure [44]: copyright 2022, Springer Nature; collagen structure of skin [45]: copyright 2023, Springer Nature; full skin structure [46]: copyright 2022, John Wiley and Sons.

Biomimetic tactile sensors, which mimic the mechanisms and microstructures found in plants, animals, and humans, offer enhanced tactile perception that can even surpass human capabilities, as illustrated in Figure 1 [36,37,38,39,40,41,42,43,44,45,46]. The Mimosa possesses unique microstructures on the surface of its leaves, such as irregular patterns and protuberances, which enable it to detect even slight mechanical stimuli and efficiently convert them into biological signals. By incorporating these microstructures into the design of flexible tactile sensors, Su et al. created a film with enhanced dielectric properties, leading to improved sensitivity and responsiveness in the sensors [36]. Moreover, other plant microstructures, such as the superhydrophobic and raised micromorphology of lotus leaf surfaces [37,47], the microdome structures of rose petals [38,48], and the hierarchical microstructure of banana leaves [39], have been strategically utilized in the design of advanced tactile sensors. In addition to plant-inspired microstructures, animal-inspired microstructures, such as the cicada wing [40], the glowing suckers of the octopus [41,49], and mammalian whiskers [42,50], can also serve as inspiration for the fabrication of biomimetic tactile sensors. Furthermore, tactile sensors inspired by the structure of human skin and fingertips have been widely studied, particularly in texture recognition. Multilayered structures and microstructured surfaces of these sensors mimic the dermis and epidermis of human skin, significantly enhancing their sensitivity [44,45,46,51,52]. Leveraging these biomimetic microstructures, tactile sensors effectively detect subtle physical changes, enabling precise, high-resolution identification of tactile signals.

Previous reviews have systematically discussed biomimetic sensors based on different sensing mechanisms. Herein, we focus on biomimetic sensors for tactile signal perception. We introduce the sensing mechanisms of biomimetic tactile sensors, including piezoresistive, capacitive, piezoelectric, and triboelectric effects. Then, we discuss the microstructures of biomimetic tactile sensors and their fabrication methods. Next, we explore the system architecture design of biomimetic tactile sensing systems. Subsequently, we present the recent applications of biomimetic tactile sensors in texture recognition, human health monitoring, and HMI. Finally, we analyze the challenges and future developments of biomimetic tactile sensors, aiming to provide a reference for future research in this field.

## 2. Sensing Mechanisms of Biomimetic Tactile Sensors

Biomimetic tactile sensors are classified into four types based on their sensing mechanisms: piezoresistive, capacitive, piezoelectric, and triboelectric. These mechanisms transform physical stimuli, such as pressure and strain, into corresponding electrical signals [24,53,54]. Each sensing mechanism has its own advantages and disadvantages in practical applications [55,56]. In this section, we will introduce these biomimetic tactile sensors according to their different mechanisms, along with the materials used for their sensitive layers. It is worth noting that the mechanisms involved in tactile perception also include optical [57,58], magnetic [59,60], and electrical impedance tomography [61,62]. However, since these mechanisms are not commonly used in biomimetic tactile sensing, they will not be further discussed.

### 2.1. Piezoresistive Sensing Mechanism

**Figure 2:**
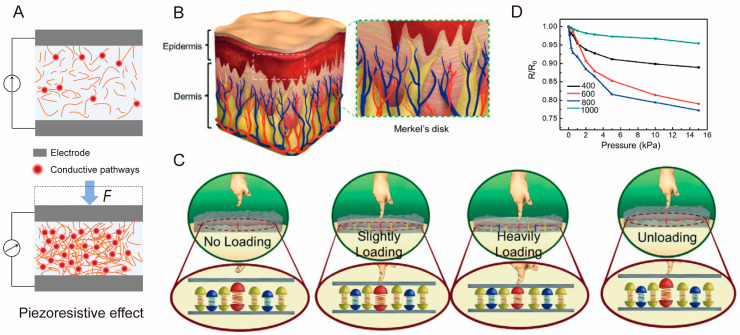
(**A**) Schematic illustration of piezoresistive effect. Piezoresistive sensor inspired by epidermis [80]: (**B**) Biological microstructure of human epidermis. (**C**) Sensing mechanism of piezoresistive sensor with spinosum microstructure. (**D**) Sensitivity comparison of piezoresistive sensor with different surface roughness. Copyright 2021, John Wiley and Sons.

The piezoresistive effect refers to the phenomenon whereby a material’s resistance changes in response to applied pressure [63,64]. When pressure is applied to the surface of a piezoresistive tactile sensor, the sensitive layer, made of piezoresistive materials or composites, deforms accordingly. This deformation alters the material’s microstructure, such as lattice spacing or conductive pathways, leading to a change in resistance. The resulting resistance variation can be measured using an electrical circuit and converted into an electrical signal, enabling the detection of physical stimuli (Figure 2A).

Common piezoresistive materials include conductive polymers, carbon-based materials, metal nanomaterials, and conductive composite materials [65,66]. Among these, conductive composite materials are typically created by mixing conductive fillers, such as metal nanoparticles, metal nanowires, carbon black, graphene, and carbon nanotubes (CNTs) [67,68], with soft polymer matrices like polydimethylsiloxane (PDMS) [69], thermoplastic polyurethane [70,71], and polyurethane [67,72]. However, the incompatibility between metal nanoparticles and polymers, as well as the dispersion of carbon-based materials in polymers, presents challenges that need to be addressed during the preparation process [73].

With the advancement of smart medicine, the demand for improved sensitivity and biocompatibility in piezoresistive sensors has grown significantly. The utilization of biocompatible materials and the design of biomimetic structures within these sensors have emerged as highly effective strategies to address these challenges. Biomimetic piezoresistive sensors not only demonstrate high sensitivity and biocompatibility, but also offer advantages such as low cost, simple structure, durability, and a wide measuring range [55,56,74,75]. Specifically, these sensors operate by the piezoresistive effect, whereby external pressure induces changes in electrical resistance directly, enabling precise signal conversion with high sensitivity. Furthermore, the use of simple and durable structures, often based on single-layer or composite materials, ensures cost-effectiveness. Biomimetic designs inspired by natural systems further enhance these sensors’ mechanical flexibility and adaptability, resulting in a wide measurement range and improved compatibility with biological tissues.

However, a major limitation of these sensors is their susceptibility to temperature variations, which necessitates the implementation of temperature compensation methods or the application of high-temperature-resistant coatings [76,77]. Furthermore, the sensitivity significantly decreases in the high-pressure range, leading to these sensors having a narrow linear range. Consequently, achieving both linearity and high sensitivity across a broad pressure range remains a considerable challenge.

Numerous studies have shown that optimizing high-performance piezoresistive materials and designing biomimetic microstructures can significantly enhance the piezoresistive effect in sensors [78]. Furthermore, the internal resistance of sensors is affected by factors such as the number of contact points, the contact area, and the distribution of conductive paths [79]. For instance, Zhu at al. developed a biomimetic tactile sensor inspired by the structure of the human epidermis (Figure 2B) [80]. The sensor is characterized by a rough surface that mimics the microspined layer found in the epidermis, which is critical for achieving high sensitivity. As shown in Figure 2C, under no external pressure, there are few contact points between the two sensitive layers. However, as the applied pressure increases, the number of contact points grows, leading to a reduction in output resistance. This behavior is a special type of piezoresistive effect, as it is driven by changes in the number of conductive pathways, rather than intrinsic changes in material resistivity. As the roughness increases within an appropriate range, the number of contact points also increases (Figure 2D), thereby enhancing the piezoresistive effect.

### 2.2. Capacitive Sensing Mechanism

**Figure 3:**
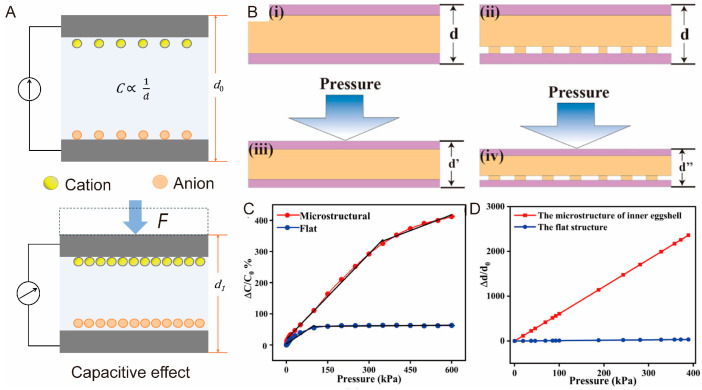
(**A**) Schematic illustration of capacitive effect. (**B**) Working principles of flat and microstructured sensors [102]. (**C**) Sensitivity comparison between flat and microstructured sensors. (**D**) Compression displacement comparison between flat and microstructured sensors. Copyright 2021, Springer Nature.

Capacitive effect refers to the conversion of pressure changes into variations in capacitance [81,82], as shown in Figure 3A. When an external force is applied to a capacitive tactile sensor, the dielectric layer deforms, altering the capacitance and thus modifying the electrical signal. Capacitance reflects the sensor’s ability to store electrical charge, which depends on the area of the conductive layers, the distance between them, and the properties of the dielectric material [83,84].

Dielectric materials include silicone rubbers, polymers with high dielectric constants, composite dielectric materials, and ionic gels [85,86]. Silicone rubbers, such as PDMS and Ecoflex, are commonly used in flexible capacitive sensors due to their flexibility, high elasticity, low dielectric loss, and excellent processability [87,88]. High dielectric constant polymers, including polyvinylidene fluoride (PVDF) [89] and poly(vinylidene fluoride-trifluoroethylene) (P(VDF-TrFE)) [90], polyimide [91], and epoxy resins [92], are also widely used. Additionally, composite dielectric materials, which are elastomers filled with high-dielectric-constant fillers, are a promising choice [93]. For example, incorporating fillers such as titanium dioxide (TiO_2_), barium titanate (BaTiO_3_), or CNTs into PDMS can significantly enhance its dielectric constant [94].

Unlike piezoresistive sensors, capacitive sensors exhibit temperature independence, which arises from the stability of dielectric materials and the inherent design of capacitive structures. This ensures consistent performance in fluctuating environmental conditions. Moreover, the high linearity of capacitive sensors results from their proportional relationship between capacitance and displacement, which minimizes signal distortion [95,96]. This characteristic makes them particularly well suited for applications requiring high resolution and a wide dynamic range [95,97,98]. Additionally, capacitive sensors offer the advantage of low power consumption. This is attributed to their operation based on the capacitive effect, which requires minimal current flow, making them highly energy-efficient.

However, similarly to normal capacitive sensors, biomimetic capacitive sensors are also not suitable for environments with strong electric and magnetic fields, due to their susceptibility to external electromagnetic interference [99]. Furthermore, parasitic capacitance and the fringe effect in device miniaturization pose limitations to their further application [100]. Incorporating biomimetic microstructures into sensor design represents a feasible strategy to address these challenges [101]. For example, He at al. reported a biomimetic capacitive sensor with a microstructured dielectric layer (Figure 3B) [102]. Compared with the sensor without a microstructure, the fabricated sensor exhibited improved sensitivity due to greater displacement (Figure 3C,D). As a result, the enhanced signal-to-noise ratio effectively reduced parasitic capacitance.

### 2.3. Piezoelectric Sensing Mechanism

**Figure 4:**
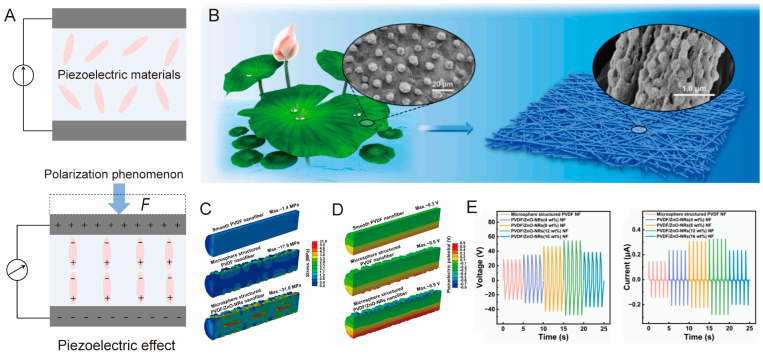
(**A**) Schematic illustration of piezoelectric effect. (**B**) Piezoelectric sensor inspired by lotus leaf [110]. Finite element analysis: stress distribution (**C**) and piezoelectric potential distribution (**D**) of three nanofibers. (**E**) Output voltage and current of PVDF/ZnO nanofibers with different ZnO contents. Copyright 2025, Elsevier.

The piezoelectric effect in tactile sensors occurs when certain materials generate an electric charge in response to mechanical stress (Figure 4A). When a piezoelectric material is subjected to an external force, its internal lattice structure deforms, resulting in changes in volume or shape [103]. This deformation arises from the displacement of electric dipoles within the piezoelectric material, leading to polarization and the generation of surface charge [103,104]. The generated charge is typically transient, decaying once the external force is removed.

Piezoelectric materials include piezoelectric polymers such as PVDF [105], piezoelectric ceramics like lead zirconate titanate and BaTiO_3_ [106,107], and two-dimensional piezoelectric materials such as molybdenum disulfide and zinc oxide (ZnO) nanosheets [108,109]. Additionally, composites that combine piezoelectric nanomaterials with flexible polymers represent another viable option.

For example, Wu at al. presented a biomimetic piezoelectric sensor by designing microspheres on PVDF/ZnO nanorods (NRs) nanofibers (Figure 4B) [110]. As shown in Figure 4C,D, stress is mainly concentrated on the microsphere-structured PVDF/ZnO-NRs, thus enhancing the piezoelectric output of the sensor (Figure 4E). Generally, biomimetic piezoelectric sensors leverage the piezoelectric effect to provide a wide dynamic range, exceptional durability, reliable performance, and self-powering capabilities [111,112]. However, the piezoelectric effect inherently limits their performance in certain scenarios. Specifically, due to their reliance on dynamic changes in mechanical stress to generate measurable signals, piezoelectric sensors are not well suited for measuring low-frequency or static signals [113].

### 2.4. Triboelectric Sensing Mechanism

Triboelectric tactile sensors typically consist of two layers made from materials with different electron affinities, which indicate their varying capacities to attract electrons. Among triboelectric materials, certain polymers exhibit strong electron affinity, such as polytetrafluoroethylene [114], PVDF [115], and fluorinated ethylene propylene [116]. In contrast, polymers with weak electron affinity, such as PDMS, polyethylene, and polyamide, tend to donate electrons [117]. This characteristic makes them suitable for use as positive layers in triboelectric pairs.

**Figure 5:**
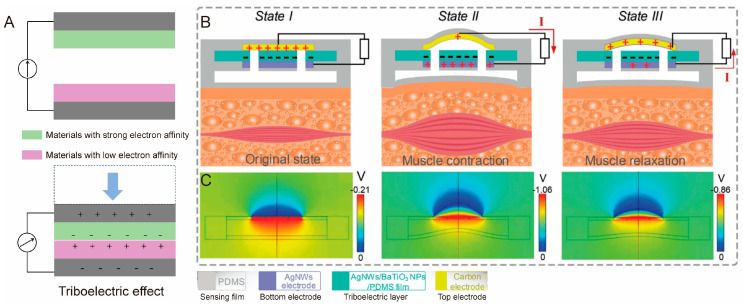
(**A**) Schematic illustration of triboelectric effect. Charge behavior of biomimetic triboelectric sensor during three muscle states (**B**) and corresponding potential distributions (**C**) [119]. Copyright 2021, John Wiley and Sons.

Upon contact and separation, electron transfer occurs between these materials, resulting in a potential difference between the electrodes (Figure 5A) [118]. To equilibrate this potential difference, electrons flow back, thereby generating a current. The repetitive mechanical actions of contact and separation result in periodic charge fluctuations between the electrodes. By monitoring these changes, the force applied to the sensor can be precisely measured.

The integration of biomimetic microstructure with the triboelectric effect provides numerous opportunities to enhance the performance and expand the functionality of triboelectric sensors. For example, Zhou at al. presented a biomimetic triboelectric sensor inspired by the structure of a frog’s mouth for microvibration monitoring of the masseter muscle in real time [119]. As shown in Figure 5B, in the first state, negative charges accumulate on the triboelectric layer and positive charges on the top electrode after several contact–separation cycles. In the second state, as the top electrode moves away from the triboelectric layer due to muscle contraction, the potential difference between the two layers increases gradually. This leads to electron flow from the top electrode to the bottom electrode. In the third state, the top electrode gradually returns to the initial state due to muscular relaxation, causing a decrease in the potential difference. Consequently, an opposing current is generated in the external circuit. The potential distributions at each of these stages are shown in Figure 5C. The biomimetic triboelectric sensor significantly enhances the functionality of human–machine interface systems.

Biomimetic triboelectric sensors offer several advantages, including being lightweight, compact, energy-efficient, and exhibiting high sensitivity and fast response times. However, similarly to biomimetic piezoelectric sensors, biomimetic triboelectric sensors are primarily suitable for dynamic sensing. Additionally, environmental factors, such as humidity and temperature variations, pose significant challenges to their stability and reliability. For example, humidity can influence the surface charge density by altering material properties or promoting charge dissipation, while temperature variations may affect the triboelectric charge generation process [120,121].

## 3. Microstructure Design for Biomimetic Tactile Sensors

The design of biomimetic microstructures is essential for the development of biomimetic tactile sensors, as it significantly affects the sensor’s sensitivity, accuracy, and overall performance. By imitating the complex structures found in nature, these microstructures enable the sensors to replicate the sensory capabilities of biological systems.

**Figure 6:**
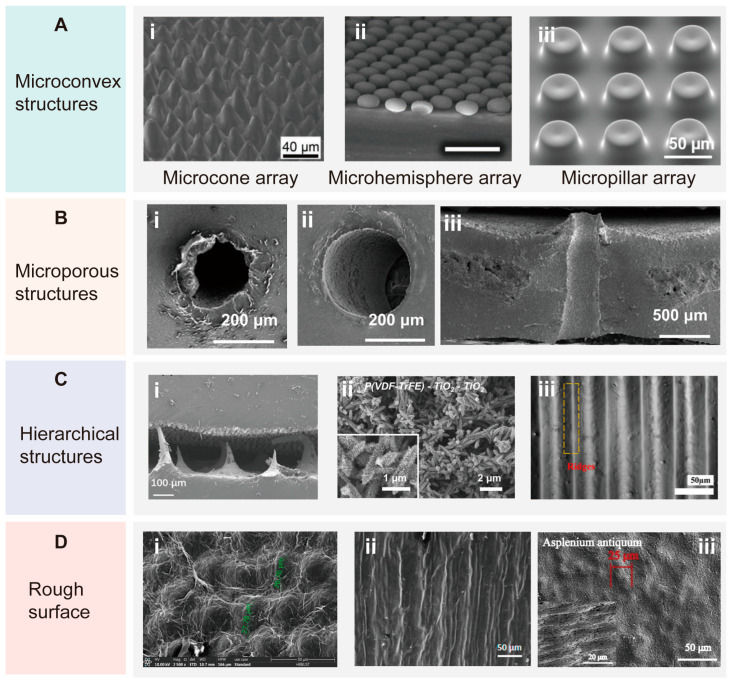
SEM images of four common bioinspired microstructures. (**A**) Microconvex structures, including (**i**) microcone array [124], (**ii**) microhemisphere array [125], and (**iii**) micropillar array [126]. Copyright 2019, John Wiley and Sons; copyright 2019, John Wiley and Sons; copyright 2023, Elsevier. (**B**) Microporous structures: (**i**) modified triboelectric layer with microporous structures; (**ii**) encapsulation layer with microporous structure; (**iii**) cross-section image of microporous structure of E-skin [127]. Copyright 2024, John Wiley and Sons. (**C**) Hierarchical structures: (**i**) combinations of microdome structure and microcone tip structure; (**ii**) hierarchical distribution of “branch-seed-spininess” structure, using different materials; (**iii**) combinations of macroscale base with serpentine shape and layer with wrinkled/cracked structure [128,129,130]. Copyright 2022, John Wiley and Sons; copyright 2023, Elsevier; copyright 2024, American Chemical Society. (**D**) Rough surfaces inspired by rose petals (**i**), banana leaves (**ii**), and cercis chinensis (**iii**) [39,48,131]. Copyright 2023, Springer Nature; copyright 2017, American Chemical Society; copyright 2021, Elsevier.

Biomimetic microstructures, drawing inspiration from natural systems, replicate the intricate mechanisms found in biological organisms, thereby leading to the development of tactile sensors with superior sensitivity, adaptability, and responsiveness. These microstructures are predominantly classified into four distinct categories: microconvex structures, microporous structures, hierarchical structures, and rough surfaces (Figure 6).

Microconvex structures are designed with protrusions that concentrate external pressure on specific regions, facilitating the detection of subtle pressure changes. The presence of multiple protrusions enables the simultaneous sensing of tactile information at different locations, making them well suited for multipoint touch applications [122,123]. Some representative microconvex structures include microcone array, microhemisphere array, and micropillar array. As shown in Figure 6A, the bioinspired design of the conical structure draws from the microstructure of the conical array found on the surface of the natural plant (Calathea zebrina). In Yao’s study, the design incorporates interlocking microstructures on the triboelectric layers of the sensor, which are strategically aligned to enhance its performance. When pressure is applied, the top and bottom layers come into contact, thereby causing an increase in the frictional contact area between them. This unique structural arrangement maximizes the contact surface between the triboelectric layers under external pressure, leading to more efficient transfer of electrons. As a result, the triboelectric effect is significantly enhanced, thereby improving the sensor’s sensitivity and responsiveness to mechanical stimuli [124]. Bui at al., inspired by the toe pads of tree frogs, developed a microhemisphere array, a structural design that facilitates large contact areas and allows for significant deformation even under small compressive forces [125]. This innovation greatly enhances the performance of triboelectric nanogenerators by optimizing the efficiency of charge transfer. Zhu et al., drawing inspiration from the setae of geckos and the suckers of octopuses, designed micropillar arrays featuring frustum-shaped conical micropits (FCMs) [126]. These micropillars were fabricated using Direct Laser Writing Lithography and then transferred onto PDMS through soft lithography. The resulting FCM structure demonstrated an adhesion strength of 1.2 MPa, which is 3.7 times higher than that of Flat Punch Micropillars and 2.1 times higher than that of Circular Conical Micropits, significantly improving the adhesive properties of the sensor. This strong adhesion can enhance the stability of the sensor’s contact with the measured object, helping to reduce measurement errors caused by poor or uneven contact.

Microporous structures typically contain micrometer- to nanometer-sized pores that can be arranged regularly or randomly. Liu at al. developed a three-dimensional microporous array that was inspired by human skin (Figure 6B) [127]. The good breathability and high sensitivity of this sensor can be attributed to its microporous structure, which enhances air permeability and increases surface area. A larger surface area provides more contact points with the external environment, thereby enhancing the detection of pressure changes. Additionally, the microporous structure facilitates the efficient transmission of water vapor and air, which is essential for maintaining comfort when the device is in contact with the skin. This breathability helps to regulate moisture and heat balance, enabling effective gas exchange between the human body and the environment.

Hierarchical structures are also a common approach in microstructure design for tactile sensors. These structures are often created by combining multiple microstructures (Figure 6C). For example, asymmetric complementary structures are fabricated by mimicking the distinctly different natural surface structures of rose petals and mulberry leaves (Figure 6C-i), thereby increasing the internal contact area [128]. Moreover, inspired by the “branch-seed-spininess” three-dimensional hierarchical structure of the cocklebur, Niu at al. constructed an electronic skin (E-skin) named the P(VDF-TrFE) fiber-TiO_2_ pillar-TiO_2_ thorn structure (Figure 6C-ii) [129]. This structure emulates the unique and complex hierarchical architecture of the cocklebur, which includes randomly distributed “branches” (P(VDF-TrFE) fibers), densely growing “seeds” (TiO_2_ nanopillars), and vertically aligned “spines” (TiO_2_ nanothorns). Such a design facilitates the amplification and concentration of localized stress. Furthermore, inspired by the intricate structure of butterfly wings, researchers utilized the layer-by-layer printing capabilities of 3D printing technology to fabricate densely packed, orderly inverted V-groove structures [130]. Wrinkles and cracks are formed between these inverted V-grooves through a prestretching process, creating a 3D hierarchical micro/nanostructure with a base that mimics the intricate patterns found on butterfly scales (Figure 6C-iii). The inverted V-grooves act as strain concentration points, controlling and accelerating strain propagation, which enhances the sensitivity of the sensor. Additionally, the wrinkles and cracks gradually unfold and expand during strain application, leading to a notable change in resistance and further improving the sensor’s sensitivity.

Furthermore, sensors with rough surfaces can be designed by using microstructures derived from natural organisms as templates. As shown in Figure 6D, inspired by the micro–nano structure of rose petals, Zhao at al. successfully fabricated a flexible capacitive sensor based on a Ag nanowires/PVDF composite dielectric layer and a PDMS rough-surface electrode [48]. Nie et al., drawing inspiration from the hierarchical microstructure and microcracks of banana leaves, enhanced the surface roughness of the device [39]. Tang at al. constructed E-skin based on the micro–nano structures of various plant leaves, including thalia dealbata, asplenium antiquum, nerium oleander and cercis chinensis [131]. Compared to traditional flexible sensors [132,133], these innovative microstructures in the E-skin significantly enhance sensitivity by amplifying mechanical signals generated under low pressures. This makes them particularly effective for detecting subtle changes, such as gentle touches or minute vibrations. Moreover, these microstructures effectively minimize mechanical hysteresis, resulting in a rapid response time. This feature is especially beneficial for dynamic applications like motion detection or human–machine interfaces. Additionally, the biomimetic microstructures possess the remarkable ability to distribute stress evenly. This characteristic helps to reduce the risk of material fatigue or failure with repeated use, ultimately enhancing the durability of E-skin.

## 4. Fabrication Methods for Biomimetic Microstructures

As surface science, materials science, and engineering have advanced, various traditional and advanced technologies have emerged for the precise fabrication and efficient production of microstructures. Common techniques employed in the fabrication of microstructures include the template method, photolithography, chemical vapor deposition (CVD), and 3D printing. These techniques are also utilized for the fabrication of bioinspired microstructures.

**Figure 7:**
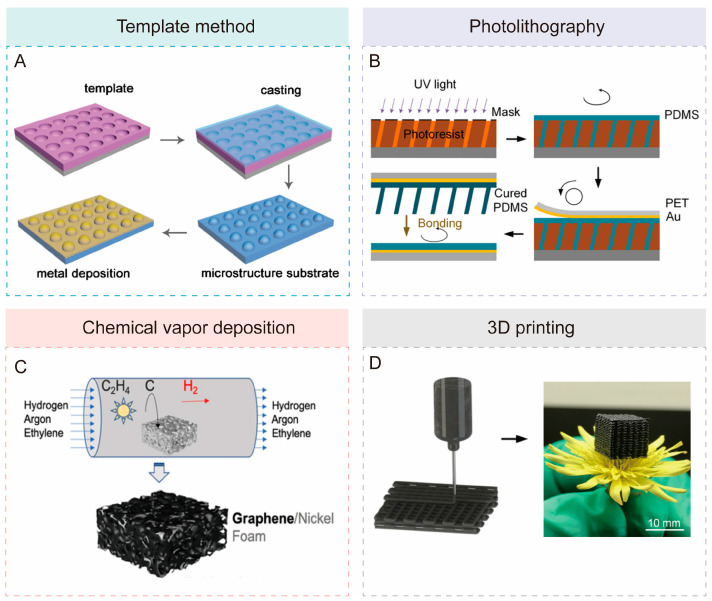
Schematic illustrations of four fabrication methods: (**A**) template method [137], (**B**) photolithography [141], (**C**) CVD [147], and (**D**) 3D printing [154]. Copyright 2018, The Royal Society of Chemistry; copyright 2019, American Chemical Society; copyright 2021, Elsevier; copyright 2020, American Chemical Society.

The template method is a technique that employs a specific template to reproduce the targeted microstructures (Figure 7A) [134,135,136,137]. Initially, elastic materials are utilized to fabricate flexible templates. Subsequently, the microstructures are replicated onto the surface of the casting material, and finally, the templates are removed through demolding processes. Depending on the requirements, further deposition of metallic materials may be considered. In addition to using customized templates, bioinspired microstructures can also be obtained by directly employing natural templates from the environment. However, both customized and natural templates may be prone to errors such as deformation and distortion during the replication process [55].

The photolithography process uses photoresists and masks to create precise microstructures [138,139,140]. As shown in Figure 7B [141], a mask with a microporous array pattern is brought into contact with the photoresist-coated substrate. The assembly is then exposed to ultraviolet (UV) light to transfer the pattern onto the photoresist. Subsequently, PDMS is poured onto the microstructured photoresist, followed by the application of a poly(ethylene terephthalate) (PET) foil with a Au layer. After curing, the PDMS layer, along with the PET foil, is peeled off from the template, forming a structured PDMS layer with micropillar arrays. The photolithography technique offers extremely high resolution, typically at the micron or even nanometer scale, enabling the fabrication of highly complex biomimetic structures. Furthermore, photolithography is widely employed in the semiconductor industry, benefiting from mature processes and equipment that support the large-scale production of bioinspired microstructures with high reproducibility [142]. However, photolithography faces several challenges, such as high equipment and material costs, as well as complex processing. Additionally, it is primarily suited for silicon-based materials and certain polymers, which limits its applicability to novel flexible materials and biomaterials [55,143,144].

CVD technology offers unique advantages in the fabrication of microstructures, primarily due to its precise control over the deposition of materials onto substrates [145,146,147]. As shown in Figure 7C, CVD involves the reaction of gaseous precursors in a chamber to grow graphene on nickel foam [147]. In the field of bioinspired microstructure fabrication, this technology can be utilized to fabricate artificial whiskers with uniform morphology and high-density alignment [148,149]. However, precise control over processing parameters, including temperature, pressure, and gas flow rate, presents technical challenges. Additionally, the slow deposition rate limits its applicability in large-scale production [150,151].

Three-dimensional printing technology, also known as additive manufacturing, constructs 3D structures by sequentially depositing materials in layers (Figure 7D) [152,153,154]. For example, Guo at al. developed a flexible tactile sensor with a reconfigurable flea bionic structure utilizing 3D printing technology [155]. This emerging manufacturing method shows promise in overcoming the challenges associated with traditional techniques for accurately replicating complex biomimetic structures [156]. Multi-material 3D printing enables the creation of layered structures with mechanical gradients, allowing integrated materials to exhibit multiple sensing mechanisms at the same time [157]. However, challenges persist in balancing resolution, cost, and speed, as well as in fabricating multi-scale structures [158].

## 5. System Architecture Design for Biomimetic Tactile Sensing System

**Figure 8:**
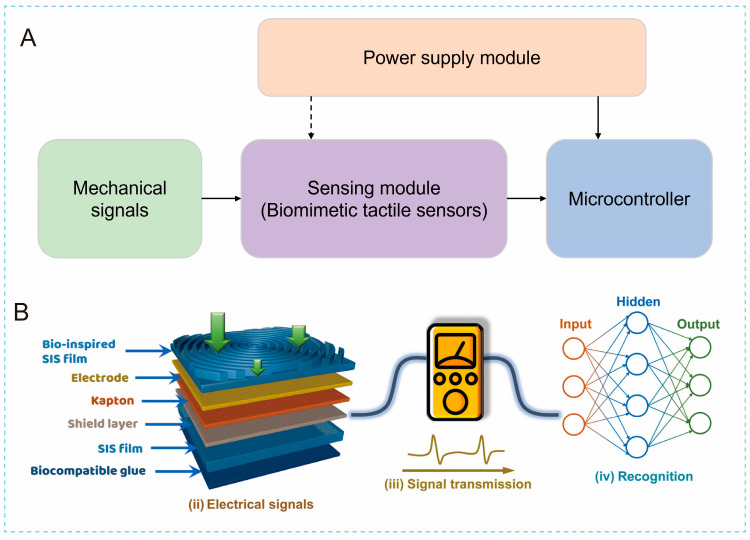
(**A**) Schematic illustration of fundamental architecture of biomimetic tactile system. (**B**) Design of bioinspired tactile perception system utilizing fingerprint-inspired E-skin, signal transmission system, and artificial neural networks [169]. Copyright 2021, Elsevier.

Due to the integration of multiple hardware modules, designing a sensing system is complex and requires balancing the trade-offs among these modules [159,160,161]. Similarly to traditional sensing systems, a typical biomimetic tactile sensing system consists of a power module, a sensing module, and a signal processing module (Figure 8A) [24,162,163]. The power module, such as batteries, is responsible for supplying the necessary power to the other modules in the system. The sensing module is composed of biomimetic tactile sensors that detect external mechanical signals. These sensors can be integrated into clothing, directly attached to the skin surface, or installed on intelligent robots. The signal processing module can convert the raw signals acquired by sensors into data suitable for subsequent analysis and decision-making.

In the signal processing module, the Analog-to-Digital Converter (ADC) is responsible for converting the analog signals from the sensor into digital signals that can be processed by the microcontroller or processor. The resolution and sampling rate of the ADC are important factors that affect the accuracy and speed of the signal processing. A higher resolution and sampling rate can provide more accurate and detailed information about the tactile signals, but they also increase the power consumption of the system. The Digital-to-Analog Converter is used to convert the digital signals back into analog signals for driving the display module or other analog devices. Furthermore, filters are used to remove noise and interference from the tactile signals [164,165]. Different types of filters, such as low-pass filters, high-pass filters, and band-pass filters, can be used to select the desired frequency range of the signals. Human perception of roughness primarily relies on low-frequency vibratory signals. Based on this characteristic, Oddo at al. employed a low-pass filter to extract low-frequency signals, enabling accurate encoding and recognition of surface roughness [166]. In contrast, Li at al. used a high-pass filter in their study to enhance the detection fidelity of bioelectric signals from the human body, demonstrating the filter’s efficacy in biomedical signal acquisition [167]. Notably, the design of the filters affects the power consumption of the system as well. A more complex filter may provide better noise reduction, but also consumes more power. Moreover, feature extraction is an important step in signal processing for biomimetic tactile sensors. The methods for feature extraction include manually specified properties, unsupervised feature learning, and machine learning [54]. Manually specified properties rely on expert knowledge, and unsupervised feature learning can automatically discover structures within data, while machine learning methods automatically extract and select features through model training, offering greater adaptability and generalization capabilities. For instance, Sun at al. performed a visual clustering analysis of 16 types of table tennis action signals coupled with a machine learning model, demonstrating the ability to differentiate between various motion signals [168].

For more advanced tactile sensing systems, additional components such as a wireless transmission module, a user interface, and software may also be included. The wireless transmission module enables communication with external devices, such as smartphones or computers, using common wireless technologies like Bluetooth, WiFi, Near Field Communication, or Radio Frequency Identification. This module ensures data encryption and secure transmission to protect user privacy. Moreover, the user interface acts as a medium for system interaction, featuring elements such as physical buttons, touchscreens, and voice control. Additionally, software is responsible for implementing the system’s functionality, including data processing algorithms, user interface design, and wireless communication protocols. When designing each module, it is crucial to consider factors such as compatibility, size, shape, and flexibility to ensure seamless integration into wearable devices.

For example, Figure 8B shows a triboelectric sensing system inspired by fingerprints [169]. When a tactile sensor makes contact with and slides across a textured surface, such as sandpaper or Braille, the mechanical signals are transformed into electrical signals. These electrical signals are subsequently captured by a voltage measurement system. These signals not only reveal the texture characteristics of the surface, but also exhibit repeatability, providing a solid foundation for accurate recognition by an artificial neural network algorithm. Notably, the sensor operates without an external power source, as it directly converts mechanical energy into electrical energy. The data processing module is primarily responsible for collecting, preprocessing, extracting features, and classifying the electrical signals. Finally, the output results of the data processing module (i.e., the results of texture recognition) are displayed through the display module.

## 6. Applications of Biomimetic Tactile Sensors

Due to their remarkable performance, including high sensitivity, rapid response, multimodal perception, and stability, biomimetic tactile sensors have been successfully applied in various fields, such as intelligent robotics, food analysis, object detection, biomedicine, environmental interaction, and HMI [101,170,171,172,173,174]. The integration of biomimetic microstructures has significantly enhanced the sensing performance of tactile sensors, improving their adhesion and hydrophobic properties. These advancements enable the sensors to process fine tactile data, demonstrating environmental perception capabilities comparable to, or even surpassing, those of biological systems. Here, we mainly summarize the applications of biomimetic tactile sensors in texture recognition, human health detection, and HMI.

### 6.1. Texture Recognition

Biomimetic tactile sensors play a crucial role in enhancing the recognition of materials and textures. Inspired by the tactile systems of natural organisms, most of these sensors replicate the touch perception of fingerprints, allowing for the accurate identification of object features. They can operate through single sensing mechanisms [169,175,176,177,178], or by combining dual or triple sensing mechanisms [173,179], making them highly effective for detecting and analyzing the material type and texture of objects. Recent studies have shown that biomimetic texture recognition technology is generally inspired by animal fur and human skin or fingerprints.

#### 6.1.1. Animal-Inspired Tactile Sensor for Texture Recognition

**Figure 9:**
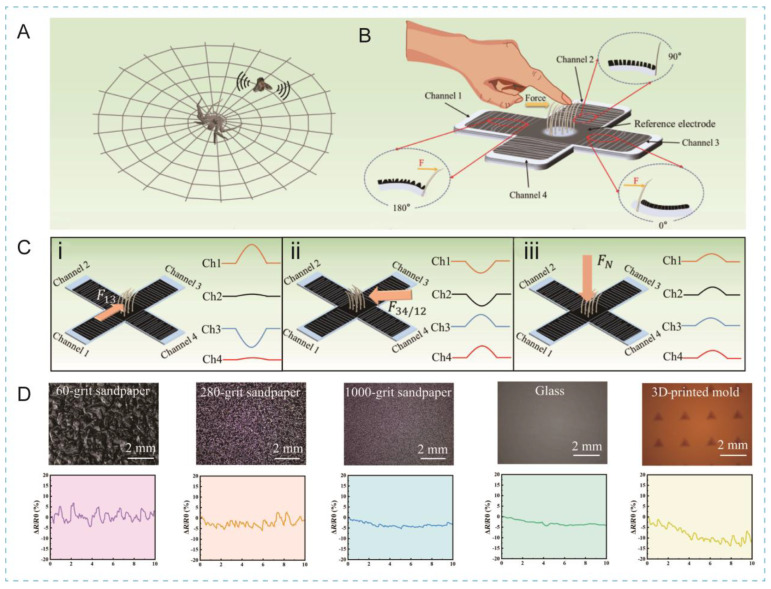
Biomimetic piezoresistive tactile sensors for texture recognition. (**A**) Highly sensitive slit organs and bristles found in spiders. (**B**) Schematic illustration of sensor’s structure. (**C**) Illustrations showing signal outputs from 4 channels under different forces: F_13_ (**i**), F_34/12_ (**ii**), and normal force (**iii**). (**D**) Relative resistance change signal output (**bottom**) of sandpaper with different textures (**top**) [43]. Copyright 2023, The American Association for the Advancement of Science.

Although traditional piezoresistive sensors are easy to fabricate and operate, they typically lack the ability to resolve complex mechanical parameters such as force direction and surface texture. Inspired by spiders (Figure 9A), Zhang at al. recently developed a piezoresistive tactile sensor (Figure 9B) featuring a synergistic design that integrates microcrack and bristle structures with a cross-shaped configuration to detect both the magnitude and direction of mechanical stimuli [43]. As shown in Figure 9C, distinct signal outputs from the four channels allow for the differentiation of shear and normal force directions. For example, when a shear force is applied from channel 1 to channel 3 (denoted as F_13_), the resistance in channel 3 decreases while the resistance in channel 1 increases, enabling precise direction detection. This capability was demonstrated in surface texture evaluation, where the sensor scanned sandpaper of varying roughness (Figure 9D). As the roughness decreased from 60-grit to 1000-grit sandpaper, the amplitude of the output signal decreased correspondingly. Moreover, an artificial neural network was used to classify the output signals, achieving a high accuracy of 96.2% for texture recognition based on 200 groups of data from five different textures.

Most existing piezoresistive sensors exhibit sensitivities ranging from a few to several tens of N^−1^, whereas high-performance capacitive and triboelectric sensors can achieve even higher sensitivities [180,181,182,183]. The biomimetic approach in this study not only provides high sensitivity (25.76 N^−1^), but also addresses the limitations of previous tactile sensors by offering direction-resolving capability.

#### 6.1.2. Human-Inspired Tactile Sensor for Texture Recognition

Human skin is highly sensitive and can detect a wide range of mechanical stimuli, including pressure, vibration, and tensile strain. It also has the ability to recognize complex textures through the combination of multiple sensory receptors. Notably, Skedung et al. demonstrated that human fingertips are capable of dynamically detecting surface structures at the nanoscale by combining materials science with psychophysics, providing new possibilities for the application of biomimetic tactile sensing technologies [184]. By mimicking the microstructure of human skin, tactile sensors can be designed to effectively detect multiple mechanical stimuli. Moreover, these sensors can overcome some limitations of natural skin, including the inability to provide quantitative data and the susceptibility to individual factors like background information, expectations, and psychological state [185,186].

For example, Lee at al. developed a multimodal E-skin by integrating triboelectric, piezoelectric, and piezoresistive sensing mechanisms [179]. The piezoresistive effect detects changes in static pressure and strain, while the triboelectric effect responds to vibrations caused by contact friction. Additionally, the piezoelectric effect is sensitive to strain. This combination enables the sensor to effectively detect subtle mechanical changes across various scenarios, thereby enhancing its overall sensitivity.

**Figure 10:**
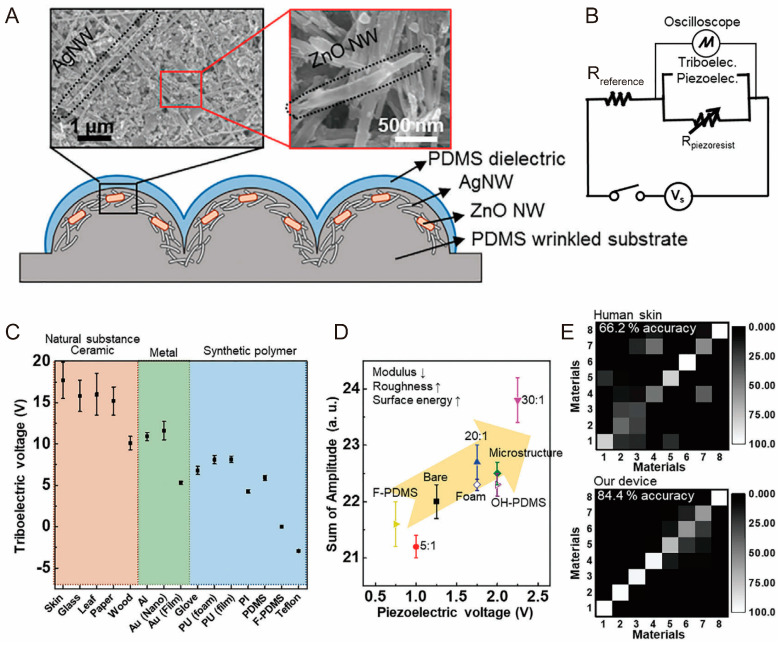
Biomimetic triboelectric, piezoelectric, and piezoresistive tactile sensors for texture recognition. (**A**) Device structure of E-skin; (**B**) circuit diagram of E-skin; (**C**) triboelectric voltages generated by E-skin upon contact with various materials; (**D**) piezoelectric voltage signals versus sum of amplitude for frequency to distinguish different textures (an upward arrow indicates an increase, and a downward arrow indicates a decrease); (**E**) confusion matrix for classification task in PDMSs with eight different textures [179]. Copyright 2021, John Wiley and Sons.

The E-skin is made from a wrinkled silicon elastomer, combined with a hybrid nanomaterial of silver nanowires and zinc oxide nanowires. Additionally, the hybrid nanomaterial is coated with a thin elastomeric dielectric layer, which features high surface roughness that mimics human fingerprints, as shown in Figure 10A. Figure 10B illustrates the basic circuit configuration of the E-skin, which includes circuits for measuring both the triboelectric/piezoelectric and piezoresistive mechanisms. For the triboelectric and piezoelectric mechanisms, measurements are conducted under open-circuit conditions, with the device electrodes directly connected to an oscilloscope to capture voltage changes. In contrast, for the piezoresistive mechanism, measurements are performed under short-circuit conditions, with an additional reference resistor and power supply used to monitor voltage changes. This circuit configuration detects the mechanical signals applied to the sensor, thereby improving the accuracy of distinguishing between different stimuli. However, in practical applications, completely separating the mechanical signals can be difficult, potentially affecting recognition accuracy.

Figure 10C–E demonstrate the performance of the multimodal E-skin in material and texture recognition. Different materials possess distinct surface potentials that influence the contact electrification process, ultimately affecting the generated triboelectric voltage. Accordingly, the E-skin can identify different materials based on the triboelectric voltage induced upon contact, including natural substances, ceramics, metals, and synthetic polymers (Figure 10C).

In addition, the piezoelectric voltage and the sum of amplitude for frequency are related to the surface energy, roughness, and modulus of the PDMS samples. As the surface energy and roughness increase and the modulus decreases, both the piezoelectric voltage and the sum of the amplitude will increase (Figure 10D). This phenomenon is attributed to the increased adhesion force between the E-skin and the contacted object, which leads to greater deformation of the E-skin, resulting in a higher piezoelectric voltage and an enhanced frequency response. Ultimately, the E-skin has proven to successfully distinguish PDMS samples with different textures. As shown in Figure 10E, the E-skin can identify eight different PDMS textures with a classification accuracy of 84.4%, surpassing the accuracy of human skin (62.2%). This recognition capability broadens the sensor’s applications in fields such as robotic grasping, object sorting, and environmental monitoring, thereby enhancing its versatility.

### 6.2. Human Health Detection

Human health detection is crucial for maintaining overall well-being and preventing potential health issues. This process involves the tracking of physiological signals such as body temperature, pulse, blood pressure, and respiratory rate. It also includes human motion monitoring to assess physical health and promote posture correction. The engineering design of biomimetic microstructures offers a novel strategy for human health detection systems.

#### 6.2.1. Human Motion Monitoring

**Figure 11:**
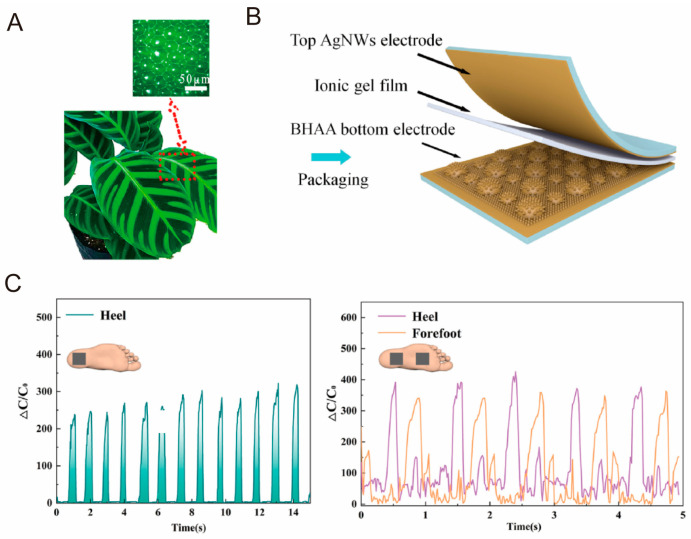
Applications of biomimetic tactile sensors in human motion monitoring. (**A**) Calathea zebrina leaf and its microcones; (**B**) structure of capacitive tactile sensor inspired by Calathea zebrina leaf; (**C**) sensors attached to heel and forefoot for gait monitoring [136]. Copyright 2022, Elsevier.

Several studies have demonstrated that incorporating porous structures is an effective method for enhancing the sensitivity of capacitive sensors [187,188]. However, the formation of micropores in the dielectric layer reduces the dielectric constant, complicating signal processing. To address this challenge, Wong and co-workers developed a capacitive tactile sensor inspired by the Calathea zebrina leaf (Figure 11A) for gait monitoring [136]. Biomimetic microstructures with mini-domes and microcones (Figure 11B) can enhance the contact area and deformation under pressure, leading to a more significant change in capacitance. As shown in Figure 11C, the sensor achieves a wide pressure sensing range for human gait detection. When the sensor is placed on the heel, it captures the pressure variations during the heel strike phase of walking, providing a stable and uniform waveform that reflects the gait cycle. Additionally, by attaching two sensors to the sole (one on the heel and one on the forefoot), the sensor can detect the entire walking process, including the heel strike, mid-stance, and toe-off phases. The normalized signals from the sensors exhibit detailed plantar pressure variations, including stride frequency and the buffering effect with the floor. This information allows for the recognition of movement posture and provides valuable insights into gait patterns, demonstrating the sensor’s potential for gait monitoring and analysis.

#### 6.2.2. Physiological Signal Monitoring

In addition to monitoring human motion, biomimetic tactile sensors are also employed to detect physiological signals. Here, we focus on their applications in detecting pulse, blood pressure, and respiratory rate, based on the four sensing mechanisms previously discussed. Traditional sensors often lack the sensitivity required to detect weak physiological signals, such as pulse and respiration, making it challenging to capture subtle changes accurately. In practical applications, sensors are susceptible to interference from external factors, such as body movement and environmental noise, which degrade signal accuracy and stability. For instance, wearable sensors are prone to motion artifacts during physical activity, disrupting signal monitoring. Recent studies have shown that integrating biomimetic microstructures into tactile sensors can mitigate these challenges.

**Figure 12:**
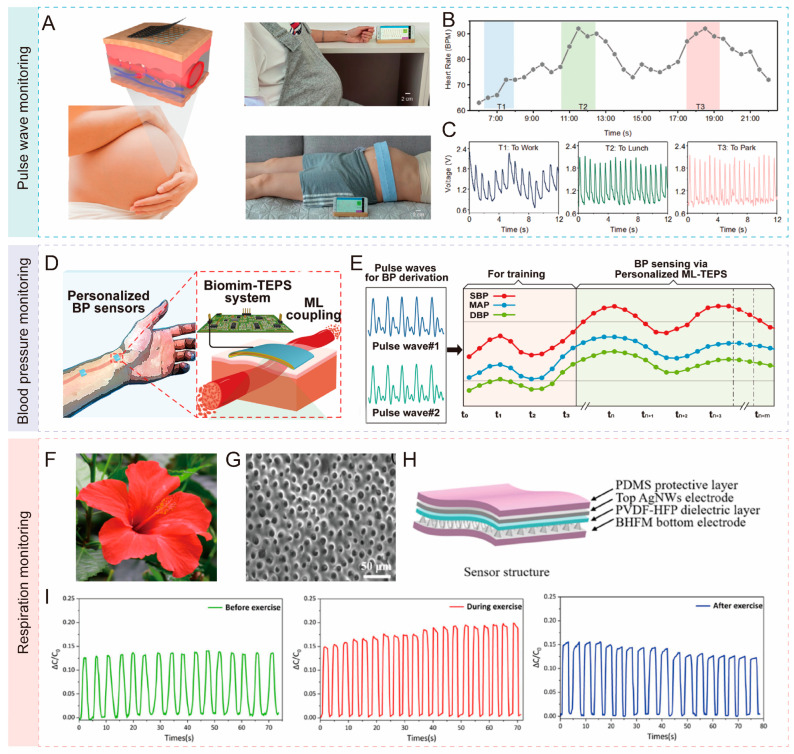
Applications of biomimetic tactile sensors in vital sign monitoring. (**A**) Biomimetic tactile sensor was worn by pregnant woman; (**B**) continuous pulse wave monitoring; (**C**) three stages of pulse wave signals acquired by tactile sensors [189]. Copyright 2024, John Wiley and Sons. (**D**) Process of acquiring blood pressure data based on pulse signals; (**E**) biomimetic tactile sensors coupled with Machine Learning algorithm for blood pressure data analysis, including Systolic Blood Pressure (SBP), Diastolic Blood Pressure (DBP), and Mean Arterial Pressure (MAP) [190]. Copyright 2023, American Chemical Society. (**F**) Photograph of hibiscus flowers; (**G**) SEM image of microstructure caves fabricated by template method; (**H**) structure of capacitive tactile sensor; (**I**) respiratory capacitance response pre-, during, and post-exercise [191]. Copyright 2024, American Chemical Society.

For example, Meng at al. developed a sensor with a honeycomb structure that can detect weak pulse signals [189]. Under the influence of external forces in different directions, the honeycomb-structured sensor effectively minimizes motion artifacts, ensuring signal accuracy and stability. The researchers also developed a health monitoring system based on this biomimetic sensor, capable of real-time pulse wave signal extraction. A pregnant woman wore a sensor during her daily activities to record her pulse waveforms and fetal movements (Figure 12A). The heart rate fluctuations of the pregnant woman, extracted from the pulse waveforms, are shown in Figure 12B. Additionally, the pulse wave signals displayed significant differences depending on her various daily activities (Figure 12C). These results demonstrate that the sensor is capable of real-time and continuous monitoring.

Additionally, continuous monitoring of blood pressure is essential for analyzing cardiovascular diseases and guiding clinical treatment. Monitoring pulse wave signals serves as an indirect method for measuring human blood pressure. Recently, Xie’s group designed a triboelectric layer for a sensor by mimicking the surface structure of cicada wings, with the aim of enhancing the sensor’s sensitivity to pulse wave signals, leading to more accurate blood pressure measurements [190]. The biomimetic sensor was attached to a human artery (Figure 12D) and successfully detected pulse wave signals. By integrating a machine learning model, the system established a personalized relationship between pulse signals and blood pressure for individual users (Figure 12E). To enable real-time signal processing and display, the group designed a micro-sized circuit board including several components: serial modules, a power module, a sensor interface, a signal processing module, a microcontroller unit, and a Bluetooth module. This circuit board converts the detected pulse signals into digital data and transmits them to a mobile device via Bluetooth for real-time recording and visualization.

Furthermore, inspired by the biomimetic microstructure of the hibiscus flower (Figure 12F,G), Lan at al. developed a biomimetic capacitive sensor. As shown in Figure 12H, the sensor incorporates a hibiscus flower-inspired microstructure on the surface of the bottom electrode, which interfaces directly with the dielectric layer. The sensitivity of the sensor with the biomimetic microstructure was approximately 4.21 times higher than that of the sensor without it. To monitor breathing, the sensor was attached to a face mask, covering the user’s nose and mouth. As the user breathed, the expansion and contraction of the chest caused pressure changes inside the mask. These pressure variations were detected by the sensor and converted into electrical signals through changes in capacitance. Therefore, the sensor can precisely measure breathing rate and intensity, providing real-time feedback on respiratory patterns. In experiments, the sensor successfully detected the subject’s breathing patterns during different physical states, as illustrated in Figure 12I. The results demonstrated that the sensor could accurately monitor respiratory rates, with 13, 17, and 14 breaths per minute recorded before, during, and after exercise, respectively.

Currently, researchers are actively exploring biomimetic microstructures, incorporating an increasing variety of natural designs into sensor technologies. This ongoing effort continues to enrich and expand the framework of biomimetic microstructures, driving advancements in sensor performance and applications in human health detection.

### 6.3. Human–Machine Interaction

As artificial intelligence emerges and develops, artificial skin will play a crucial role not only in texture recognition and health monitoring, but also in HMI. While traditional HMI systems, including touchpads and voice recognition, adequately address everyday needs, they often fall short in providing a seamless experience for individuals with speech or motor impairments, as well as those with severe health conditions [192]. In contrast, biomimetic tactile sensors offer a promising solution to these challenges, bridging the gap and enhancing the HMI experience for a wider range of users. These advanced sensors are capable of simultaneously detecting multiple tactile signals and facilitating multimodal interactions, effectively addressing the diverse needs of different user groups.

**Figure 13:**
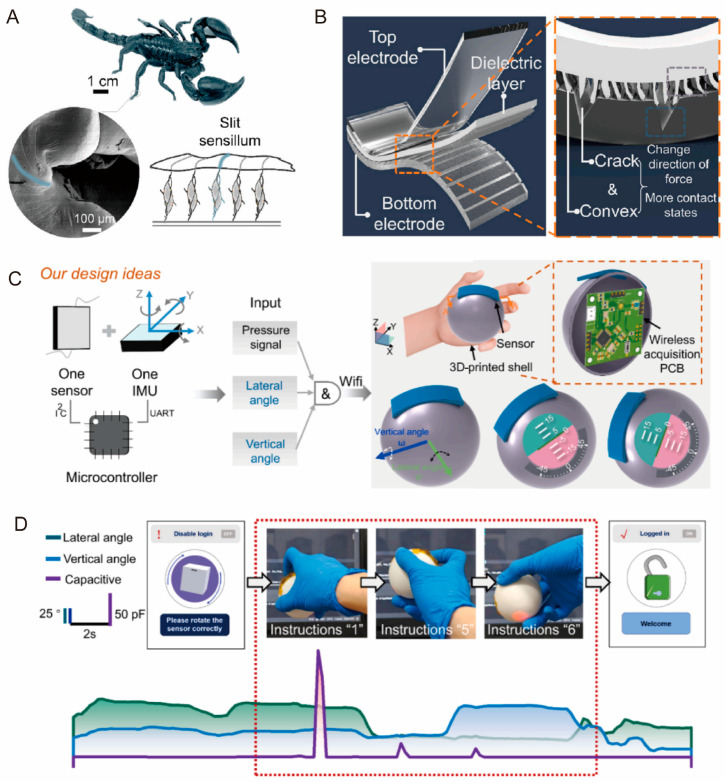
Applications of biomimetic tactile sensors in HMI [193]. (**A**) Scorpion and its slit sensillum. (**B**) Biomimetic capacitive sensor with microconvex structures and crack structures. (**C**) Novelty design ideas for sensor array. (**D**) Variation in lateral angle, longitudinal angle, and capacitance signal during unlocking process. Copyright 2025, Elsevier.

The slit sensillum of scorpions (Figure 13A) exhibits high sensitivity to weak mechanical signals. Drawing inspiration from this, Wang at al. designed a biomimetic capacitive sensor featuring microconvex structures in the dielectric layer and crack structures in the electrode layer, as shown in Figure 13B [193]. The crack structure provides a stable contact interface between ions and electrons without increasing the sensor’s overall thickness. When pressure is applied, the microstructures can change the force direction and enable the detection of multiple contact states. The fabricated single sensor can be extended into an array structure to generate multiple complex HMI signals. A traditional acquisition circuit for the sensor array typically incorporates multiplexing switches, algorithms, and optimized wiring designs. In contrast, their HMI system introduced an innovative approach by integrating the sensor array with inertial motion units, enabling the processing of more complex signals (Figure 13C). Subsequently, the sensor array was attached to the surface of a spherical plastic shell containing built-in circuit boards, enabling the system to decode spatial orientation data. This innovative setup was demonstrated by successfully unlocking a password lock through the controlled rotation of the plastic ball, as shown in Figure 13D.

## 7. Conclusions and Outlook

Biomimetic tactile sensors are becoming increasingly important in wearable electronics. The integration of biomimetic microstructures not only enhances their adaptability to diverse environments, but also introduces new functionalities. This review introduces biomimetic tactile sensors, focusing on the design of their microstructures and system architecture, their sensing mechanisms, and their fabrication methods. Moreover, their recent applications in texture recognition, human health monitoring, and HMI are discussed. Despite considerable advancements in the application of these sensors through ongoing research endeavors, several challenges still require attention.

Enhancing material/device performance remains a persistent challenge in the laboratory research stage. One effective strategy is the development of more advanced bionic microstructures to enhance sensing sensitivity. Notably, while sensitivity is a critical factor, it is not the only metric of performance. Other characteristics such as range, precision, durability, and versatility are equally crucial. For sensors utilizing composite polymers, mismatches between the property of the polymer matrix and conductive fillers can lead to stability issues. For instance, differing thermal expansion coefficients can render the device vulnerable to temperature fluctuations. Consequently, to ensure the long-term functionality of the device, it is essential to design materials and structures that can adapt to environmental fluctuations in temperature and humidity. Furthermore, addressing the signal lag during dynamic testing and mitigating potential crosstalk in high-spatial-resolution sensor arrays are critical.

In the future, the integration and large-scale manufacturing of biomimetic tactile sensors will become increasingly essential. This will require continuous updates of data processing methods and machine learning algorithms to meet user needs. Moreover, seamless integration of these sensors will be crucial, as they are expected to be incorporated into clothing and wearable medical devices for everyday use within intelligent tactile systems. For specific application scenarios, future research should focus on more nuanced discussions of trade-offs and customization. At the same time, biocompatible designs will ensure the safety of human contact, thereby paving the way for biomimetic tactile sensors to seamlessly enter daily life, and ushering in a new era of intelligent tactile technology.

## Data Availability

Not applicable.
